# P1 evoked by facial expression images is enhanced in Parkinson’s disease patients with depressive symptoms

**DOI:** 10.3389/fnagi.2024.1423875

**Published:** 2024-10-30

**Authors:** Yujia Sun, Yixiang Mo, Chunkai Peng, Qingqing Li, Zhuyong Wang, Sha Xue, Shizhong Zhang

**Affiliations:** Neurosurgery Center, Department of Functional Neurosurgery, The National Key Clinical Specialty, Guangdong Provincial Key Laboratory on Brain Function Repair and Regeneration, The Neurosurgery Institute of Guangdong Province, Zhujiang Hospital, Southern Medical University, Guangzhou, China

**Keywords:** Parkinson’s disease, event related potentials, depressive disorders, P1, biomarker

## Abstract

**Introduction:**

Depressive symptoms are most common non-motor symptoms in Parkinson’s disease (PD), which is often overlooked due to absence of rapid and objective diagnostic biomarkers. Electroencephalography (EEG)-based event-related potentials (ERPs) is commonly used to assess emotional processes. The aim of this study was to investigate changes in ERPs in PD patients exhibiting depressive symptoms and to provide a reliable biomarker for assisting in the diagnosis of PD with depressive symptoms.

**Methods:**

We conducted a case–control study involving 30 PD patients with (dPD group) or without depressive symptoms (nPD group) and 13 age matched healthy controls (HC). We recorded EEG of the patients during the emotional picture stimulation task and analyzed the difference in the early ERPs potentials (P1, N170, early posterior negativity) and their correlation with the severity of symptoms in PD patients.

**Results:**

Our results found that P1 amplitude in the occipital region of the dPD group in response to emotional faces was significantly higher than that of nPD and HC group, and it was positively correlated with severity of depressive symptoms in PD patients.

**Conclusion:**

Our study shows that facial expression-induced enhancement of P1 amplitude can be utilized as a rapid and objective indicator to screen for depressive symptoms in PD.

## 1 Introduction

Parkinson’s disease (PD) is a degenerative neurological disorder, and depressive symptoms are one of the most common non-motor symptoms of PD. The existence of depressive symptoms seriously affects the quality of life of PD patients (Su et al., 2021). At present, the screening of depressive symptoms mainly relies on structured interviews and scale evaluation, which are time-consuming, inefficient, and occasionally difficult to obtain patient cooperation (Lee et al., 2022; Ahmad et al., 2023). In addition, some patients could not complete the scale test due to the limitation of low educational level. Moreover, the overlap of depressive symptoms with some symptoms of PD can also lead to the overlook of depressive symptoms of PD (Galts et al., 2019). There are also some biomarkers associated with depressive symptoms of PD, such as blood biomarkers, inflammatory markers, and core body temperature (Suzuki et al., 2007; Santiago et al., 2016; Ramanzini et al., 2022). But some of these markers are invasive to acquire, and others may be interfered with by multiple biological factors. Therefore, the search for objectively existing non-invasive and stable biomarkers is more conducive to early screening and detection of depressive symptoms.

Emotion dysregulation is one of the core features of depression (Li et al., 2021). The realization of brain function depends on the conduction of electrical signals. Event-related potentials (ERPs) is a relatively objective and non-invasive method to measure the electrical activity of the brain during emotional and cognitive activities with high temporal resolution (MacNamara et al., 2022). ERPs in response to visual stimuli are regarded as effective tools for assessing emotional processes, which has now been widely applied in many neuropsychiatric diseases, especially in depression (Li et al., 2023). Components in the early stage such as P1, N170 and early posterior negativity (EPN) can reflect the emotional processing more directly. P1, peaking at 100–150 ms following face onset at occipital areas, is found to be associated with the rapid extraction of visual information (Batty and Taylor, 2002). N170, appearing around 170 ms at occipital areas, is sensitive to processing of emotional faces and is thought to be involved in the regulation of emotions (Hinojosa et al., 2015). Previous studies found that larger P100 and N170 amplitude can be elicited under negative stimuli in major depression disease (MDD) group as compared with healthy group (Zhang et al., 2016). In addition, another early visual ERP component, EPN with amplitude maximized at occipital regions between 220 and 300 ms, can also be modulated by emotional face and has been suggested to indicate early perceptual tagging and prioritized processing (Schupp et al., 2004; Schindler and Bublatzky, 2020). A study by Dai and Feng showed that the alterations of ERPs are induced by the magnification and sensitivity of sad stimuli in MDD patients (Dai and Feng, 2012). In conclusion, these studies indicated that ERP components can serve as an index about the changes of emotional processing in patients with depressive symptoms.

In patients with depression, some characteristic ERP components can be used as biomarkers for depression (Zheng et al., 2024). However, the pathogenesis of depression and depressive symptoms in PD patients may be different. The mechanism of disturbed emotional processing of PD-related depressive symptoms remains unclear. The purpose of this study was to compare the differences in the early components of ERPs between healthy controls and PD patients with or without depressive symptoms, to explore the pathogenesis and look for objective electrophysiological markers for depressive symptoms in PD.

## 2 Materials and methods

### 2.1 Ethics considerations

The study protocol was approved by the Ethics Committee of Zhujiang Hospital of Southern Medical University, approval number 2020-060-01. The study was conducted in accordance with the Declaration of Helsinki. Participants were told about the objective and content of the experiment. Written informed consent was obtained prior to the experiment.

### 2.2 Inclusion criteria

Patients were included in the study if the following criteria were met: (1) PD diagnosis was confirmed according to the Chinese Parkinson’s Disease Diagnostic Criteria (2016 Edition); (2) Patients were willing to cooperate with structured interviews.

### 2.3 Exclusion criteria

Patients were excluded from the study based on the following criteria: (1) cognitive dysfunction (MMSE <24 points); (2) serious (refractory) depression, anxiety, schizophrenia, and other mental diseases; (3) having a history of other neurological diseases except PD; (4) taking antidepressants; (5) having severe disorders (optic or hearing problems etc.) which may disrupt the accomplishment of clinical tests.

### 2.4 Final enrollment

**Figure 1:**
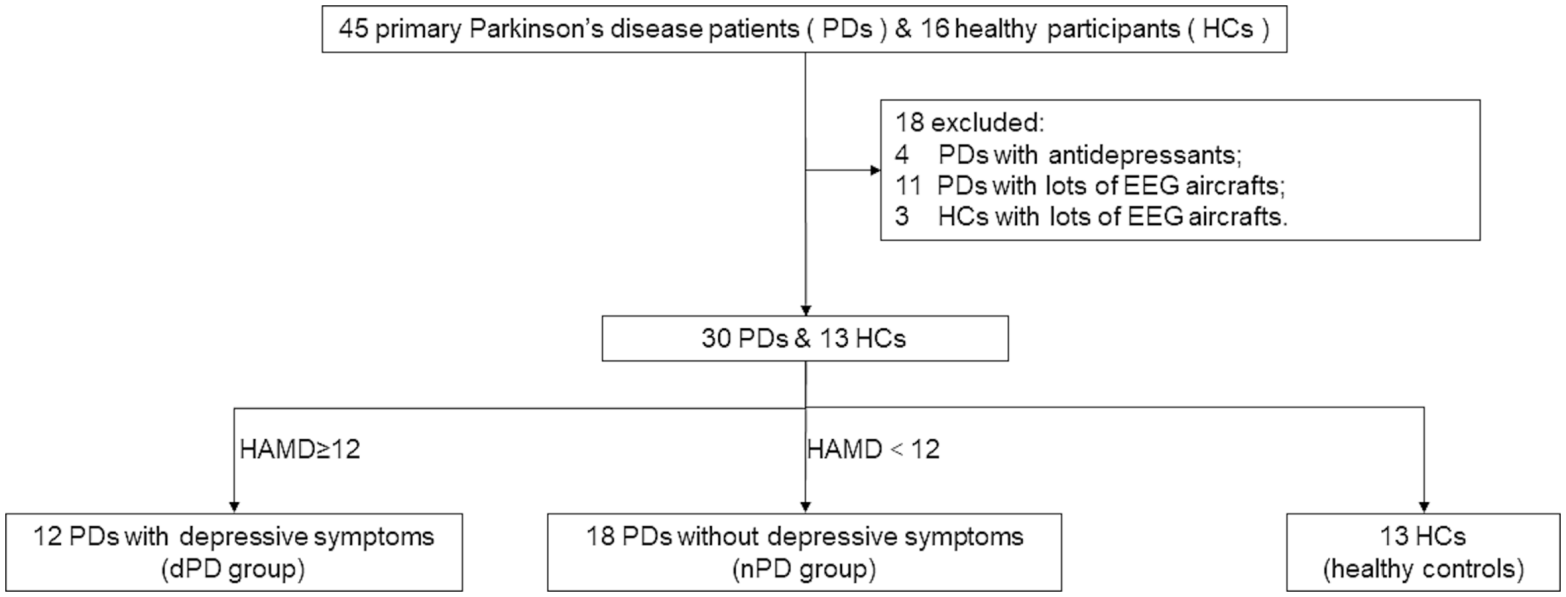
Inclusions and exclusions. PD, Parkinson’s Disease; dPD, Parkinson’s Disease with depressive symptoms; nPD, Parkinson’s Disease without depressive symptoms; HC, healthy controls.

Thirty PD patients (male: 12; female: 18; all right-handed) were finally enrolled from a total of 45 consecutive patients, from August 2020 to October 2023 in the Zhujiang Hospital of Southern Medical University. 11 patients with excessive EEG artifacts and four patients with a history of antidepressants medication were then removed. Sixteen healthy controls (HC) matched for age participated in our study, but three of them were removed from the final analysis due to the poor EEG data (Figure 1). The patients were divided into PD with depressive symptoms (dPD group, HAMD ≥12 points) and PD without depressive symptoms (nPD group, HAMD <12 points) according to the HAMD-24 score [13]. The basic clinical information of the PD patients and HC are shown in Table 1.

**Table 1:** Preoperative characteristics of PD patients and healthy controls.

Characteristic	PD with depressive symptoms	PD without depressive symptom	Healthy controls	*p*
	(*n* = 12)	(*n* = 18)	(*n* = 13)	–
Age—yr	57.75 ± 9.48	57.56 ± 6.71	61.31 ± 7.31	0.368
MMSE	26.75 ± 2.09	26.69 ± 2.02	27.31 ± 2.50	0.555
Duration of disease—yr.	8.83 ± 3.95	8.50 ± 3.96	–	0.723
LEDD— mg/day	792.08 ± 306.52	742.77 ± 319.47	–	0.677
UPDRS III off	37.17 ± 14.67	34.11 ± 15.05	–	0.587
UPDRS III on	14.96 ± 8.82	12.11 ± 6.68	–	0.415

Plus–minus values are means ± SD. There were no significant differences between groups. PD, Parkinson’s Disease; MMSE, the Mini-Mental State Examination; LEDD, Dose of levodopa or equivalent; UPDRS, the Unified Parkinson’s Disease Rating Scale.

### 2.5 Clinical assessment

Motor symptoms of PD patients were assessed using the Unified Parkinson’s Disease Rating Scale III, and the Levodopa Equivalent Daily Dosage (LEDD) was also recorded. The 24-item Hamilton Depression Rating Scale was applied for depressive symptoms evaluation. The Hamilton Anxiety Rating Scale (HAMA) was used for anxiety evaluation, and the Mini Mental State Examination (MMSE) was employed for intellectual assessment. The clinical rating of depressive symptoms was based on consensus of two senior psychiatrists. All the clinical assessments and Electroencephalogram (EEG) recordings for each participant were completed within 3 days.

### 2.6 Stimuli material and procedure

Three different kinds of emotional grayscale faces (happy, neutral and sad) were chosen as stimulus from Chinese Facial Affective Picture System. The overall recognition degree of faces chosen is set to 85%. A total of 40 non-repetitive images were selected for each type of face, and each image would be repeated three times. Each experiment consisted of 360 emotional faces, which were evenly distributed and presented to participants randomly.

**Figure 2:**
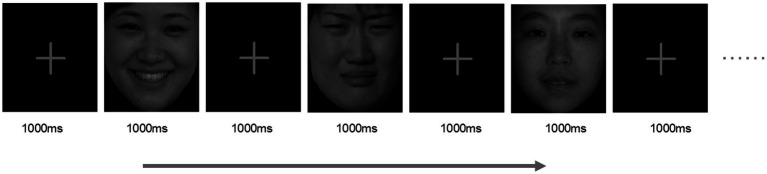
Experimental procedure. 360 images from Chinese facial affective picture system were randomly repeated in the center of the screen, including 40 happy, 40 sad and 40 neutral images which repeated 3 times each. Each image shows 1,000 ms, separated by 1,000 ms image of the cross. Reprinted with permission by Prof. Yuejia Luo, the copyright owner of Chinese Facial Affective Picture System.

All stimulus were with the same contrast and brightness displayed on a 15 inches computer screen using the E-PRIME 2.0 software package in a sound-attenuated and dimly lit room. To minimize the impact of previous stimulus, a fixation cross lasting for 1,000 ms comes out first. Then, facial stimulus continued for 1,000 ms (Figure 2). During the trials, participants were asked to gaze at different types of face stimuli carefully and avoid all activities that could produce artifacts, such as excessive eye blinks, move their bodies and make noises.

### 2.7 Data acquisition and analysis

EEG data were recorded from 28-channel Ag/AgCl electrodes according to the international 10–20 system at a sampling rate of 1,000 Hz (Neuracle, China). During recording, all electrode impedances were kept below 5 KΩ. Meanwhile, horizontal and vertical electrooculograms, which were located at the outer canthi of the eyes and at infra and supraorbital positions of left eyes, were used to record electro-ocular artifacts. For ERP analysis, the process of data was displayed by EEGLAB v2021.0 implemented in Matlab R2020a. First, all channels were re-referenced to the Cz electrode. Second, EEGs were filtered at a lowpass of 30 Hz and a highpass of 0.1 Hz and epochs containing artifacts exceeding ±100 μV were also rejected. Third, epochs were defined between 200 ms before stimulus onset and 800 ms after stimulus, and all epochs were baseline-corrected with respect to the mean voltage over the 200 ms preceding the onset of stimulus, followed by averaging in association with experimental conditions. Next, independent component analysis was performed to remove ocular and muscular artifacts. After the pre-processing procedure, ERP in all brain regions was analyzed, then the latency and peak amplitude of P100, N170 and EPN in occipital regions (Oz, O1 and O2) were extracted by ERPLAB and examined manually.

### 2.8 Statistical analysis

Dependent variables were defined as the peak amplitude and average latency time of the ERP components of interest. Responses of each participant under 3 stimuli conditions (happy, neutral, sad) were averaged. The Kolmogorov–Smirnov method was used to test the normality, and the independent sample student *t*-test or Mann–Whitney U test was used to determine the significance of the difference based on normality. Two way repeated-measures ANOVA was performed with emotion (happy, sad and neutral) as the within-subject factor, and group (nPD, dPD and control group) as the between-subject factor with Bonferroni correction. Correlation analysis between the ERP component amplitude, latency and age, LEDD, and severity of motor and non-motor symptoms of PD patients were conducted. The significance level was set at 0.05, unless mentioned.

## 3 Results

### 3.1 Characteristics of PD patients and healthy controls

No significant differences were observed for age and MMSE scores across dPD, nPD, and HC groups. Besides, no significant differences were observed for the disease duration, LEDD, UPDRSIII on and UPDRSIII off scores between dPD and nPD groups. See Table 1 for details.

### 3.2 PD patients with depressive symptoms have enhanced P1 amplitude in occipital area

**Figure 3:**
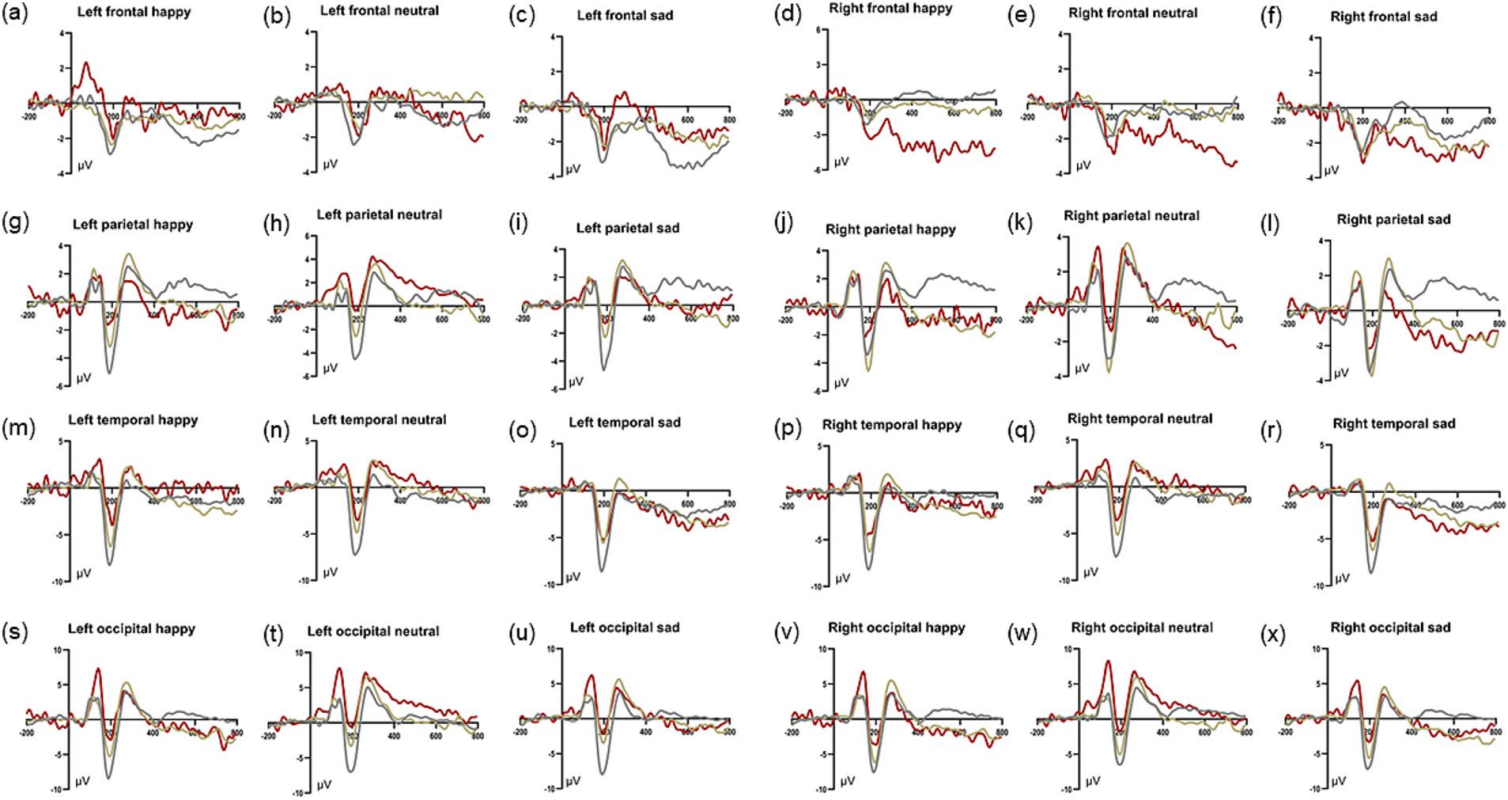
Event-related evoked potentials of whole brain for HC, nPD and dPD groups. **(a–x)** show average waveforms at left and right frontal electrodes (F3/4 and F7/8), parietal electrode (P3/4), temporal electrodes (T3/4 and T5/6), occipital electrode (O1/2) under positive **(a, d)**, neutral **(b, e)** and negative **(c, f)** stimuli in dPD, nPD and HC group, respectively.

**Figure 4:**
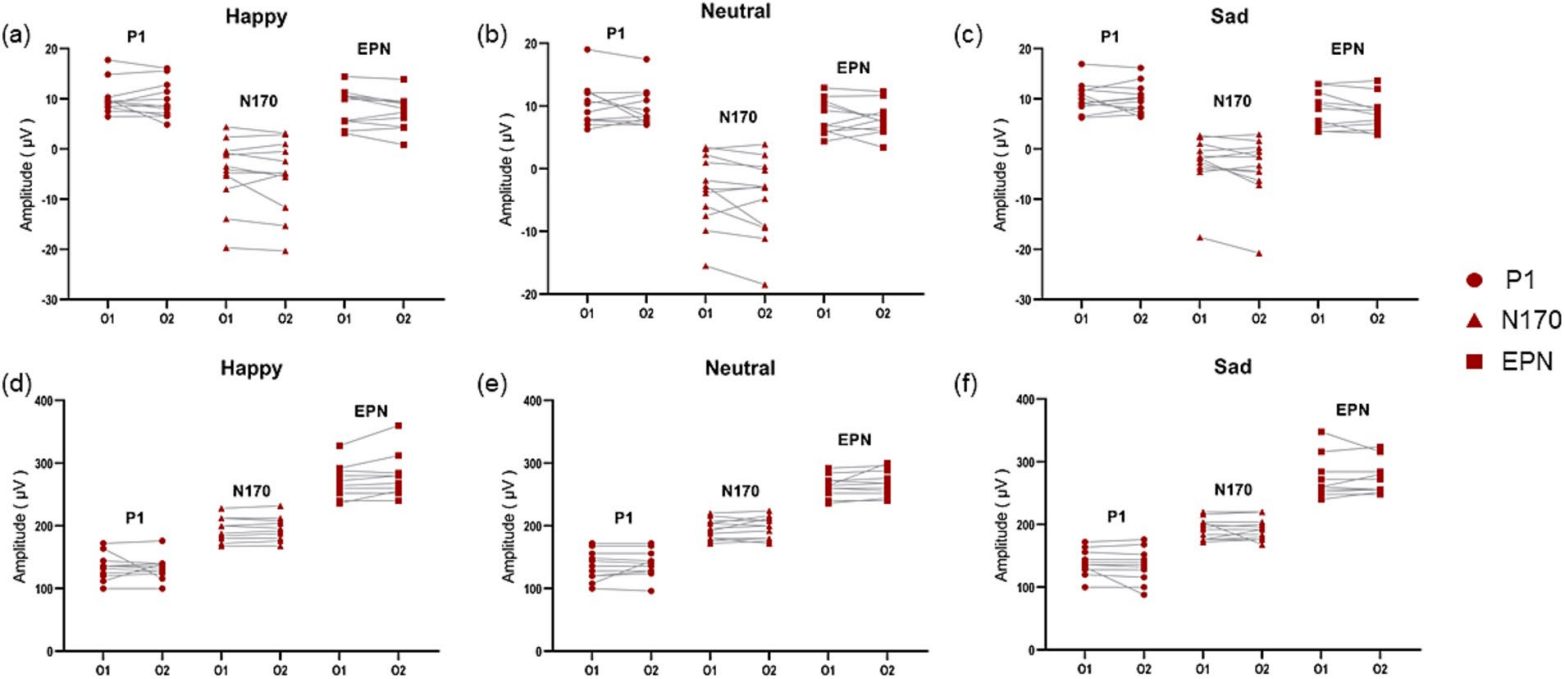
Comparison of amplitude and latency of ERPs in the left and right occipital area in PD patients with depressive symptoms. **(a–c)** Show the compare of amplitude of ERP components in O1 and O2 induced by positive **(a)**, neutral **(b)** and negative **(c)** images in dPD group. **(d–f)** Show the compare of latency of ERP components in O1 and O2 induced by positive **(d)**, neutral **(e)** and negative **(f)** images in dPD group. The circle refers to P1, the triangle refers to N170 and the square refers to EPN.

We observed ERPs in the whole brain and found that P1, N170 and EPN components could be evoked significantly in the occipital area in patients with PD accompanied by depressive symptoms (Figure 3). And there was no significant difference in P1 amplitude and latency in left (O1) and right (O2) occipital region in dPD group (Figure 4). Next, we analyzed the differences of P1, N170 and EPN components in the entire occipital region in HC, nPD and dPD groups.

For peak amplitude of P1, the main effect of group was significant (*F* (2,120) =48.153, *p*<0.001，ηp2 = 0.445). For peak amplitude of N170, the main effect of group was significant (F (2,120) =9.658, *p*<0.001，ηp2 = 0.139). For peak amplitude of EPN, the main effect of group was significant (*F* (2,120) =6.717, *p*<0.01，ηp2 = 0.101). In addition, the main effect for EPN latency of group was significant (*F* (2,120) =3.272, *p* = 0.041, ηp2 = 0.052). Except for this, there was no significant main effect of group for any latency of component. The amplitude and latency of each component had no main effect of stimuli. There was no interaction between the emotional stimuli and the groups in any component.

**Figure 5:**
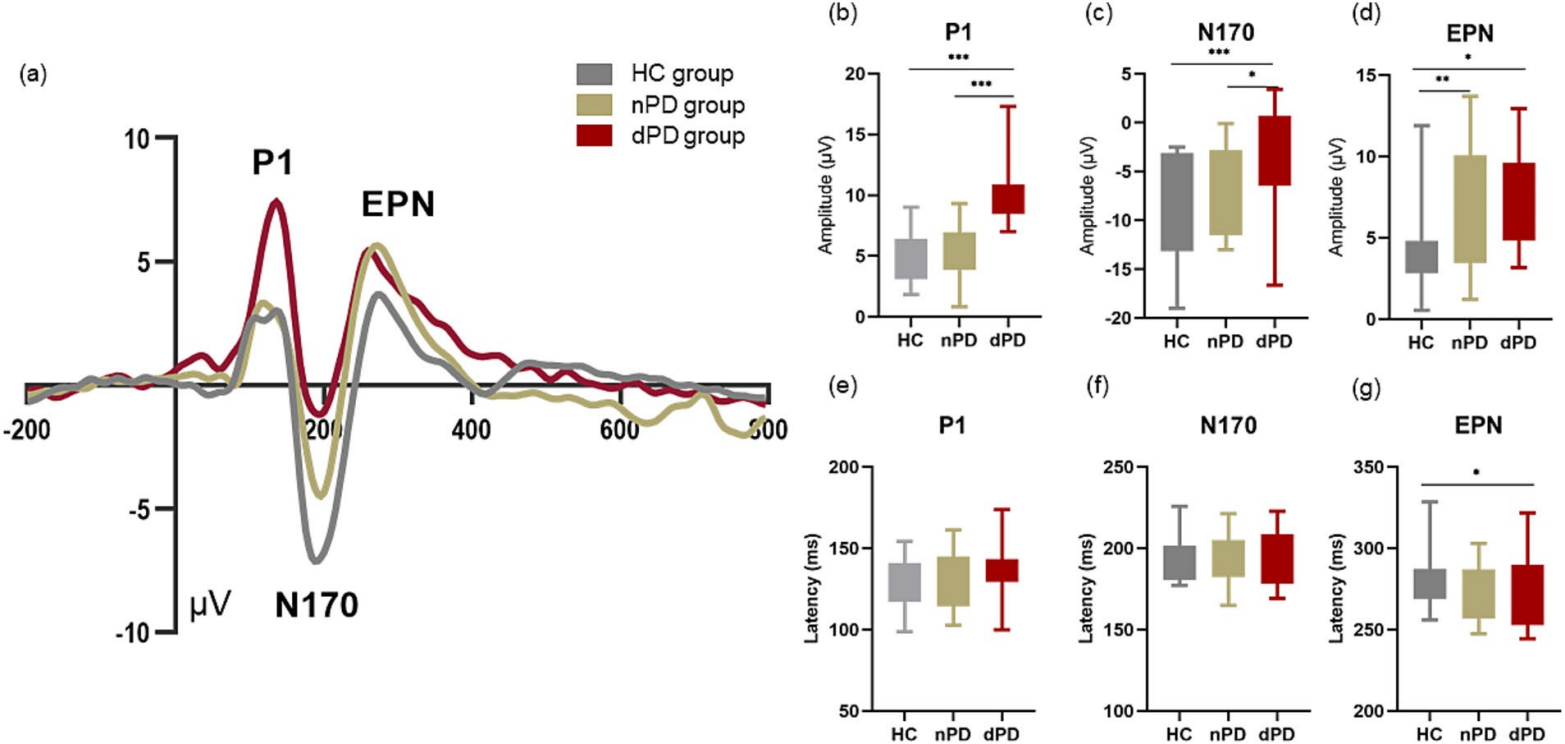
Event-related evoked potentials in occipital area. (a) Show average waveforms at occipital electrodes (Oz, O1 and O2) under in dPD (red), nPD (yellow) and HC (gray) under all type of emotional faces. The bar charts of (b–d) refers to the compare of peak amplitude of P1, N170 and EPN between the HC, nPD group and dPD group of different components. The bar charts of (e–g) refers to the compare of latency of P1, N170 and EPN between the HC, nPD group and dPD group of different components. EPN, early posterior negativity. * means *p* < 0.05, ** means *p* < 0.01, *** means *p* < 0.001.

Figure 5a shows visual evoked ERP for the HC, nPD, and dPD groups. Results of *post hoc* multiple comparisons show that peak P1 amplitude evoked by emotional images in dPD group was significantly higher than that in nPD group (Figure 5b, *p*<0.001) and HC (Figure 5b, *p* < 0.001) group, indicating an enhancement in the emotional response in the dPD group. In addition, it was found that N170 amplitude of dPD group was more reduced than that of nPD and HC group (Figure 5c, *p* = 0.045, *p* < 0.001, respectively). When it comes peak amplitude of EPN, larger EPN amplitude of nPD group and dPD group was found than that of HC group (Figure 5d, *p* < 0.01, *p* = 0.13, respectively). As for the latency (Figures 5e,f), there was no statistical difference between the components in the other groups except that the latency of EPN components in the dPD group was smaller than that in the HC group (Figure 5g, *p* = 0.48).

### 3.3 The peak amplitude of P1 in occipital area was associated with the severity of depressive symptoms in patients of PD

**Figure 6:**
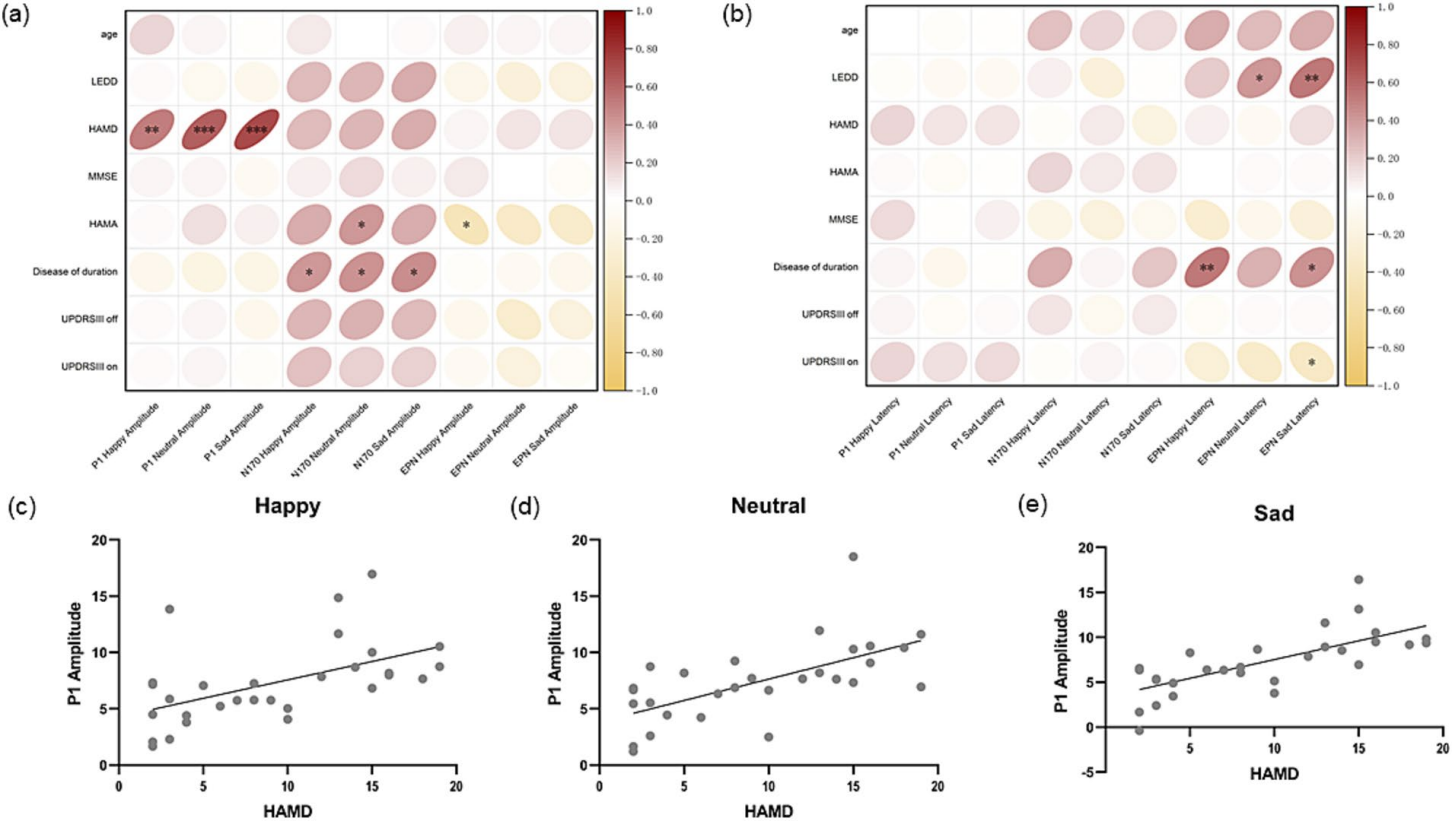
Correlation analysis of amplitude and latency of ERP components. (a) Shows the Pearson correlation coefficient between amplitude of P1, N170 and EPN induced by three different stimuli and age, LEDD, MMSE and motor symptom, non-motor symptom severity score of Parkinson’s disease at the same period. (b) Shows the Pearson correlation coefficient between latency of P1, N170 and EPN induced by three different stimuli and age, LEDD, MMSE and motor symptom, non-motor symptom severity score of Parkinson’s disease at the same period. (c) Shows P1 amplitude induced by happy stimuli is positively correlated with the HAMD score of PD patients in the same period (*Pearson’s r* = 0.529, *p* < 0.01). (d) Shows P1 amplitude induced by neutral stimuli is positively correlated with the HAMD score of PD patients in the same period (*Pearson’s r* = 0.634, *p*<0.001). (e) Shows P1 amplitude induced by sad stimuli is positively correlated with the HAMD score of PD patients in the same period (*Pearson’s r* = 0.714, *p* < 0.001). LEDD, levodopa equivalent daily dose; HAMD, Hamilton depression scale; MMSE, Mini-Mental State Examination; HAMA, Hamilton anxiety scale; UPDRS, the Unified Parkinson’s Disease Rating Scale; EPN, early posterior negativity.

To explore the correlation between ERP components and clinical symptoms in more detail, Pearson correlation was used to analyse the ERP component amplitude (Figure 6a) and latency (Figure 6b) induced by three kinds of emotions in all PD patients with their age, duration of disease, LEDD, severity of motor symptoms, and severity of anxiety and depressive symptoms, respectively. It was found that peak P1 amplitude induced by happy (Figure 6c), neutral (Figure 6d) and sad (Figure 6e) images were all positively correlated with the severity of depressive symptoms in PD patients (*Pearson’s r* = 0.529, *p* < 0.01; *Pearson’s r* = 0.634, *p* < 0.001; *Pearson’s r* = 0.714, *p* < 0.001). In addition, the amplitude of N170 components induced by happy, neutral, and sad images was significantly correlated with the duration of disease in PD patients (*Pearson’s r* = 0.427, *p* = 0.019; *Pearson’s r* = 0.445, *p* = 0.014; *Pearson’s r* = 0.432, *p* = 0.017), and the amplitude of N170 components induced by neutral emotions was also significantly correlated with the severity of anxiety symptoms in PD patients (*Pearson’s r* = 0.405, *p* = 0.027). EPN amplitude induced by happy faces was negatively correlated with anxiety symptoms (*Pearson’s r* = −0.447, *p* = 0.015). Then we analysed the correlation between P1 amplitude and factor items of HAMD and found that the amplitude of P1 was mainly associated with anxiety and somatization, cognitive impairment, retardation, sleep disturbance, and feelings of despair, but not weight loss or diurnal variation (Supplementary Figure 1). In conclusion, among the early ERPs components, only the amplitude of P1 was significantly correlated with the severity of depressive symptoms in PD patients.

No correlation was found between P1 and N170 latency induced by the three emotions in occipital area and the age, duration of disease, LEDD and symptom severity of PD patients. However, EPN induced by happy (*Pearson’s r* = 0.529, *p* < 0.01) and negative (*Pearson’s r* = 0.435, *p* = 0.018) faces was significantly positively correlated with the disease duration of PD patients. EPN latency induced by neutral faces (*Pearson’s r* = 0.411, *p* = 0.027) and sad faces (*Pearson’s r* = 0.531, *p* < 0.01) was significantly positively correlated with LEDD in PD patients. EPN latency induced by sad faces was also significantly negatively correlated with UPDRSIII on score (*Pearson’s r* = −0.375, *p* = 0.045).

## 4 Discussion

In this study, emotional face images were used to explore visual evoked potentials in PD patients with or without depressive symptoms and healthy controls. We found that patients with PD accompanied by depressive symptoms had significantly elevated P1 amplitudes in the occipital area, and the peak amplitude of P1 in occipital area was significantly correlated with the severity of depressive symptoms in PD patients.

The exploration of visual-related evoked potentials can effectively understand the information processing process and the advanced functional mechanism of brain. In this study, happy, neutral, and sad faces were selected as stimuli to determine whether PD patients with depressive symptoms have an attentional bias to certain emotions. The characteristics of ERPs in different brain regions are different, which also reflects the specific processing of functions in different brain regions (Lei et al., 2021). As a first step, we analysed ERPs across the cortex. P1, N170 and EPN components can be observed in the parietal, temporal and occipital regions. We observed the most significant P1 amplitude in the occipital region of PD patients with depressive symptoms, which is also the region of the brain used to investigate P1 component in patients with depression (Zhang et al., 2016). Our study found that enhanced P1 in the occipital area in PD patients with depressive symptoms may also suggest that the occipital lobe is also one of the functional nodes involved in the mechanism of depressive symptoms in PD. P1 is the first ERP component that appears after facial expression stimulation, reflecting an unconscious and automatic processing process (Vuilleumier and Pourtois, 2007). Some researchers have compared multiple studies and found that P1 does not have an emotional effect: that is, different emotional stimuli do not affect P1 amplitude, and the increased P1 amplitude may only represent the difference between disease groups independent of the type of stimulus face (Schindler and Bublatzky, 2020). But a lot of studies argued that patients with depression show higher P1 amplitudes evoked by sad faces on ERP tests (Dai et al., 2016), which shows that depression patients pay more attention to negative faces, representing the attention bias to negative information (Zhang et al., 2016). However, this negative attention bias may only exist in patients with MDD, and for patients with subclinical depression, they show enhanced attention to positive and neutral stimuli (Dai and Feng, 2012). This means that patients with different stages of depression present and deal with emotions differently. We recruited PD patients who do not have MDD, and our results show that PD patients with depressive symptoms have enhanced attention to positive, neutral, and negative information, which may resemble a subclinical depressive state. Early identification and intervention of subclinical depressive state in PD patients is conducive to improving the quality of life of PD patients (Chuquilín-Arista et al., 2020). In our study, the peak amplitude of P1 under different types of emotional faces was significantly positively correlated with HAMD score, and it was independent of age, disease course, LEDD, motor symptoms and other factors, which could be used as an effective and reliable marker of depressive symptoms accompanied by PD.

Regarding the latency of P1 component, previous studies have shown that depression patients have a decreased latency induced by sad stimuli (Yang et al., 2011; Shao et al., 2023). However, our study did not find any difference in P1 latency between PD patients with depressive symptoms and healthy controls. This may be related to the age of the subjects. Studies have pointed out that in visual ERP test, P1 latency decreased with age (Taylor et al., 2004). It is conceivable that the neurodegenerative changes in PD disease may have an impact on the reaction time. It is possible that age and depression combined to show such results in the latency. However, this requires ERP studies in some elderly patients with depression and healthy controls to be definitive.

Previous studies on depression have suggested that patients with depression have a lower N170 amplitude than the healthy control group, suggesting the early dysfunction of processing emotional faces (Xin et al., 2020; Xin et al., 2021). Our study showed similar results, that PD patients with depressive symptoms had lower N170 amplitudes. But N170 is an ERP component that is affected by many factors. N170 is sensitive to faces and has emotional effects and the direction of face also has a regulating effect on N170 (Denefrio et al., 2017; Schindler and Bublatzky, 2020; Tso et al., 2017). First-Episode and Recurrent Major Depression also showed different results in N170 (Chen et al., 2014). Because N170 is affected by many factors (Feuerriegel et al., 2015), and PD is a multi-system disease along with complex symptoms, the amplitude of N170 in PD patients varies greatly. So even though PD patients with depressive symptoms exhibits a lower N170 amplitude, the decreased N170 amplitude may be less sensitive than P1 in suggesting PD-related depressive symptoms.

EPN was regarded as the marker of early sensation of visual stimuli, which was relevant to the emotional valence and the priority of emotional process, and is committed to more refined stimulus processing and consciousness (Schupp et al., 2004). Previous studies on depression have suggested that the latency of EPN in major depression disorder is significantly delayed and the amplitude of EPN is reduced compared with the healthy control, which suggests early attentional processing dysfunction of emotional faces (Xin et al., 2021). In addition, there are also studies on anxiety disorders suggesting that patients with anxiety disorders have stronger EPN amplitude (Yoon et al., 2016). Our results found enhanced amplitude of EPN in PD patients and we also found that the latency of EPN in occipital area was correlated to many factors, such as the duration of the disease, the equivalent dose of levodopa, and motor symptoms severity. In other words, the amplitude and latency of EPN components may also be the result of a combination of various factors like N170. However, due to the limitation of our small sample size, we may not be able to reach a conclusion with sufficient proof to conduct multi-factor regression analysis to analyse the potential mechanism behind the change of EPN. Subsequent studies with a larger sample size can be designed to further explore.

Thus, compared to N170 and EPN, P1 in the occipital area generated by emotional faces may be a considerably more sensitive indicator for PD patients with depressive symptoms. This objective, non-invasive and quickly detectable indicator can be helpful for the screening and detection of depressive symptoms. Our results may shed light on the pathogenesis of PD accompanied by depressive symptoms.

## 5 Limitations

Due to bradykinesia of PD patients, they cannot make quick keystroke responses. If they are forced to make judgments and reactions with their bodies, there can be a lot of artifact interference affecting the ERP analysis. Thus, the paradigm only focuses on the patient’s visually relevant automatic response to stimuli, but also makes the paradigm simpler and easier for the patient to cooperate with.

In addition, most of the previous studies on depression were middle-aged and young adults, but the changes in ERP components were age-sensitive. If we want to explore the mechanism of PD depression and depression, we need to include elderly patients with depression for analysis, and we plan to continue to explore in subsequent studies.

## Data Availability

The raw data supporting the conclusions of this article will be made available by the authors, without undue reservation.
