# Supplementary material for: P1 evoked by facial expression images is enhanced in Parkinson’s disease patients with depressive symptoms
Source: Front Aging Neurosci. 2024 Oct 30;16:1423875. doi: 10.3389/fnagi.2024.1423875 (PMC11557433; doi:10.3389/fnagi.2024.1423875)
Supplement: Supplementary file 1 [file Data_Sheet_1.DOCX]

## Supplementary Figures


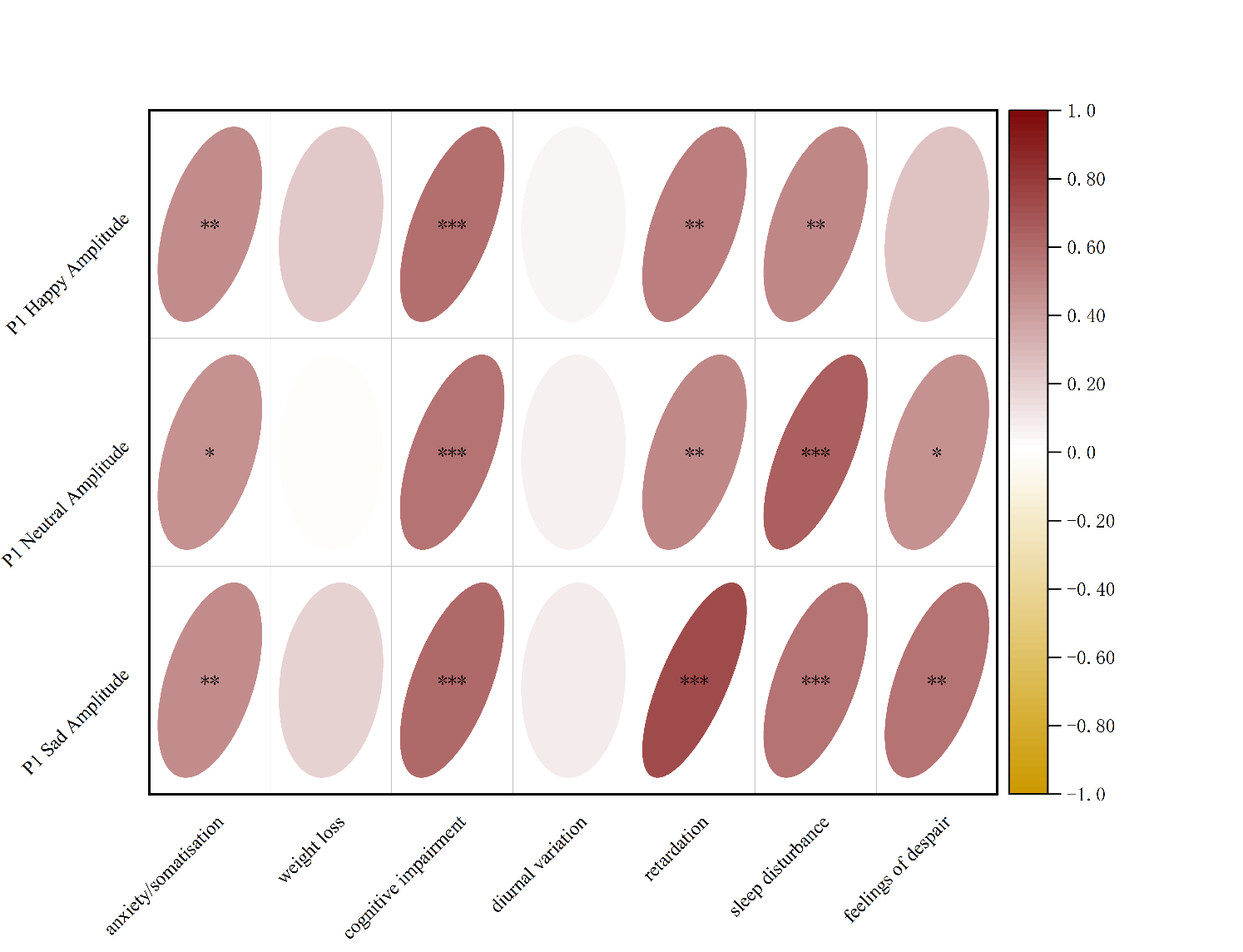


**Supplementary Figure 1.** The correlation between P1 amplitude and factor items of HAMD. Spearman correlation coefficient between amplitude of P1 induced by three different stimuli and factor items of HAMD: anxiety/somatization (items 10-13, 15 and 17), weight loss (item 16), cognitive impairment (items 2-3, 9, and 19-21), retardation (items 1, 7, 8 and 14), sleep disturbance (item 16) and feelings of despair (items 22-24) in Parkinson's disease. * means *P*＜0.05, ** means *P*＜0.01, *** means *P*＜0.001. Colors represent Spearman’s correlation values.
